# Lessons from Toxicology: Developing a 21st-Century Paradigm for Medical Research

**DOI:** 10.1289/ehp.1510345

**Published:** 2015-11-01

**Authors:** Gill Langley, Christopher P. Austin, Anil K. Balapure, Linda S. Birnbaum, John R. Bucher, Julia Fentem, Suzanne C. Fitzpatrick, John R. Fowle, Robert J. Kavlock, Hiroaki Kitano, Brett A. Lidbury, Alysson R. Muotri, Shuang-Qing Peng, Dmitry Sakharov, Troy Seidle, Thales Trez, Alexander Tonevitsky, Anja van de Stolpe, Maurice Whelan, Catherine Willett

**Affiliations:** 1 Research and Toxicology Department, Humane Society International, London, United Kingdom; 2 National Center for Advancing Translational Sciences, National Institutes of Health (NIH), Department of Health and Human Services (DHHS), Bethesda, Maryland, USA; 3 Division of Biochemistry, CSIR–Central Drug Research Institute, Lucknow, India; 4 National Institute of Environmental Health Sciences (NIEHS) and National Toxicology Program (NTP), NIH, DHHS, Research Triangle Park, North Carolina, USA; 5 Division of NTP, NIEHS, NIH, DHHS, Research Triangle Park, North Carolina, USA; 6 Unilever R&D, Safety and Environmental Assurance Centre (SEAC), Sharnbrook, Bedfordshire, United Kingdom; 7 Office of the Center Director, Center for Food Safety and Applied Nutrition, U.S. Food and Drug Administration, Maryland, USA; 8 Science to Inform LLC, Pittsboro, North Carolina, USA; 9 Office of Research and Development, U.S. Environmental Protection Agency, Washington DC, USA; 10 Systems Biology Institute, Tokyo, Japan; 11 Genomics and Predictive Medicine, John Curtin School of Medical Research, Australian National University, Canberra, Australia; 12 University of California, San Diego, School of Medicine, Department of Pediatrics/Rady Children’s Hospital San Diego, Department of Cellular and Molecular Medicine, Stem Cell Program, La Jolla, California, USA; 13 Evaluation and Research Center for Toxicology, Institute of Disease Control and Prevention, Academy of Military Medical Sciences, Beijing, China; 14 Scientific Research Centre Bioclinicum, Moscow, Russia; 15 Research and Toxicology Department, Humane Society International, Montréal, Quebec, Canada; 16 Institute of Science and Technology, Federal University of Alfenas, Alfenas, Brazil; 17 National Center of Medical Radiological Research, Obninsk, Russia; 18 Philips Research, Eindhoven, the Netherlands; 19 Institute for Health and Consumer Protection, European Commission Joint Research Centre, Ispra, Italy; 20 Regulatory Toxicology, Risk Assessment and Alternatives, Humane Society of the United States, Washington DC, USA.

## Abstract

Biomedical developments in the 21st century provide an unprecedented opportunity to gain a dynamic systems-level and human-specific understanding of the causes and pathophysiologies of disease. This understanding is a vital need, in view of continuing failures in health research, drug discovery, and clinical translation. The full potential of advanced approaches may not be achieved within a 20th-century conceptual framework dominated by animal models. Novel technologies are being integrated into environmental health research and are also applicable to disease research, but these advances need a new medical research and drug discovery paradigm to gain maximal benefits. We suggest a new conceptual framework that repurposes the 21st-century transition underway in toxicology. Human disease should be conceived as resulting from integrated extrinsic and intrinsic causes, with research focused on modern human-specific models to understand disease pathways at multiple biological levels that are analogous to adverse outcome pathways in toxicology. Systems biology tools should be used to integrate and interpret data about disease causation and pathophysiology. Such an approach promises progress in overcoming the current roadblocks to understanding human disease and successful drug discovery and translation. A discourse should begin now to identify and consider the many challenges and questions that need to be solved.

## Introduction

The genomics era opened a door to understanding genetic changes in susceptibility to diseases, such as single nucleotide polymorphisms, gene copy number variations, and gene deletions and insertions (Zerhouni 2014). The subsequent explosion of related “omics” approaches, including transcriptomics, metabolomics, and proteomics, have provided more details of how gene regulation and protein production are implicated in human disease mechanisms.

However, many human illnesses such as cancers, diabetes, immune system and neurodegenerative disorders, and respiratory and cardiovascular diseases are caused by a complicated interplay between multiple genetic and environmental factors (Lango and Weedon 2008). The environmental counterpart to genomics is exposomics, which aims to capture an individual’s lifetime exposure to external factors (e.g., infections, environmental chemicals, drugs, radiation) measured via biomarkers in blood, urine, feces, or breath samples. It provides an opportunity to develop an environmental analog of genome-wide association studies, similarly top down and hypothesis free (Lioy and Rappaport 2011).

Another emerging omics tool is epigenomics—the study of changes in gene activity not attributable to DNA sequence alterations (e.g., DNA methylation and chromatin remodeling). Epigenetic changes including inherited effects and environmentally induced alterations are implicated in disease causation, and epigenomics is being developed in disease research. The U.S. National Institutes of Health (NIH) Roadmap Epigenomics Consortium has provided detailed human epigenomic maps to enhance studies of human disease and development (NIH Roadmap Epigenomics Consortium 2015). Epigenomics is also being explored in environmental health research with many exposures being associated with adverse health effects (Shenderov and Midtvedt 2014). These developments provide an unprecedented opportunity to add a new dimension to the study of human diseases.

The 21st century has seen these and many other pivotal advances in science and technology: Together, they offer, for the first time, the possibility of gaining a dynamic systems-level and human-specific understanding of the causes and pathophysiologies of disease (van de Stolpe and Kauffmann 2015). This understanding is a vital need, in view of current failures (Scannell et al. 2012; Kaitin and DiMasi 2011) in health research, drug discovery, and clinical translation (Collins 2011). But these developments in human-specific models and tools require a new research paradigm to unlock their full potential. We suggest it is time for a novel, overarching paradigm for medical research based on adapting and applying the transitional process underway in toxicology that includes reducing reliance on animal models, and instead emphasizing human biology and approaches based on multiscale pathways.

## Discussion

**Figure 1:**
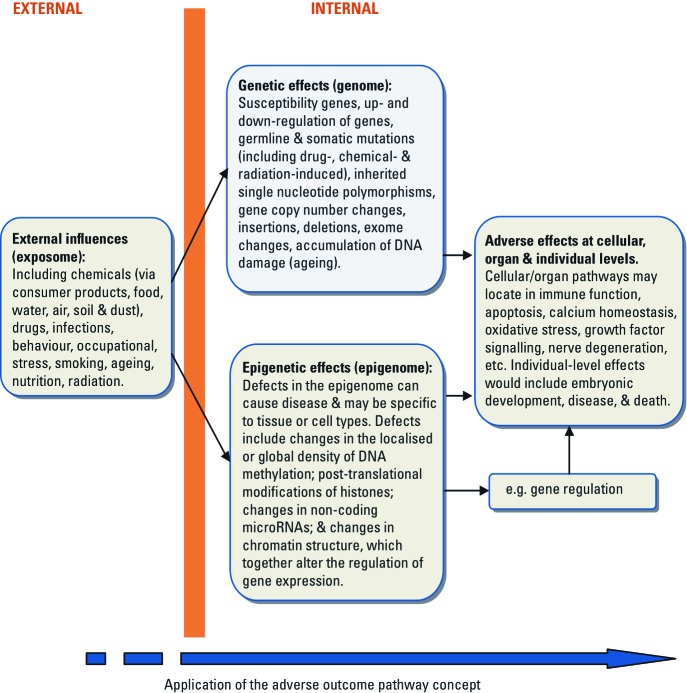
Integrating data on extrinsic and intrinsic causes of disease using systems biology provides a more comprehensive understanding of human illnesses. The adverse outcome pathway (AOP) concept links exposure, via chemical structure (or structures), the molecular initiating event, and key events, to an adverse outcome.

In future health research and drug discovery, diseases can be envisaged as the combined outcome of extrinsic causes that include many types of exposures, not just chemical exposures, and intrinsic genetic and epigenetic changes (e.g., Gohlke et al. 2009) that interact at multiple levels (Figure 1). This combined approach would provide a more coherent “big picture” by linking environmental sciences with medical research.

Some of the thinking required to develop a more comprehensive framework for understanding disease causation has already begun. Toxicologists and environmental health scientists are already devising new models that explore synergies between toxic exposures and infectious pathogens in complex diseases, exemplified by interactions between the hepatitis B virus and aflatoxin in liver cancer (Birnbaum and Jung 2010).

*A new medical research paradigm.* To maximize the value of advanced models and technologies, we believe that a new paradigm is needed for fundamental research into human diseases and for drug discovery. The focus should move decisively away from preclinical animal studies and overly simplistic cell models toward a systems biology framework to integrate new types of scientific data, such as from omics, novel human-specific *in vitro* models, and clinical studies. Such a framework would help enable a comprehensive and dynamic understanding of disease causation and pathophysiology.

A concept that systematically describes links between causes of disease and outcomes could be repurposed from 21st-century toxicology. Since the publication of the U.S. National Research Council (NRC) report calling for a new paradigm (NRC 2007), a transition in toxicology has been underway, actively supported by U.S. regulatory and research agencies both from environmental and medical arenas (Collins 2011), as well as by the European Union [Scientific Committees on Health and Environmental Risks (SCHER) et al. 2013]. The focus in toxicological research turned first to understanding toxicity pathways––the normal cellular processes involving genes, proteins, and small molecules that lead to adverse human health effects when significantly perturbed by chemical toxicants (NRC 2007).

The notion of the cell-level toxicity pathways described in the NRC report (2007) has already been extended to the broader concept of adverse outcome pathways (AOPs), thereby addressing the sequence of changes between the molecular initiating event (e.g., a chemical binds to a cell receptor) and adverse outcomes at the molecular, cellular, organ, organism, and population levels. An AOP is a standardized way to describe concisely the critical mechanisms of toxic effects and is enabling the emergence of a new predictive toxicology paradigm [Organisation for Economic Co-operation and Development (OECD) 2012]. This paradigm contrasts with classical toxicology where so-called apical toxicity end points are studied in a series of animal tests for different kinds of toxic effects [e.g., cancer, reproductive and developmental toxicity, or skin allergy (sensitization)]. However, this traditional black box approach sheds little light on the underlying pathways of toxicity. Rather, it merely presents an end result that is not easily accessible to deeper analysis or understanding.

**Figure 2:**
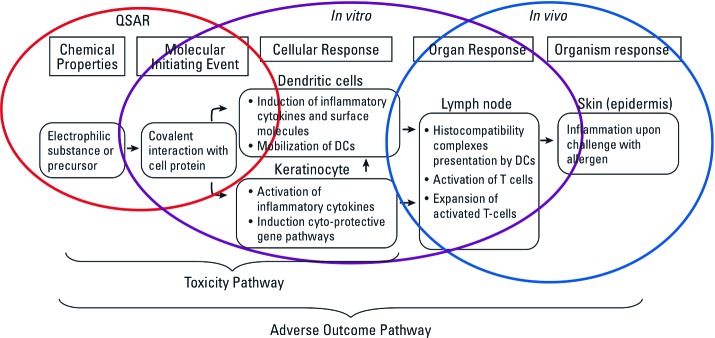
Diagram showing different pathways concepts, including the well-characterized adverse outcome pathway (AOP) for chemically induced skin allergy, from chemical structure through molecular initiating event, key events and adverse outcome. DCs, dendritic cells; QSAR, quantitative structure–activity relationships. Reprinted from Encyclopedia of Toxicology, Vol. 1, 3rd ed. Adverse outcome pathways: development and use in toxicology, pp. 95–99, 2014, with permission from Elsevier.

Because of its potential to contribute to deeper knowledge-based human and environmental health assessments, the AOP concept is now established as a comprehensive framework at the OECD to support its international regulatory programs on chemical toxicology (OECD 2013). The OECD has published the first well-characterized AOP, describing chemical potential for causing skin allergy (OECD 2012) (Figure 2), and many others are under development (e.g., Vinken et al. 2013) and review. An essential component of the OECD program is the AOP Knowledge Base (AOP-KB; http://www.aopkb.org) that facilitates scientific collaboration on an international scale to aid both the development and evaluation of AOPs.

*Repurposing the AOP concept for human health research.* We now suggest a novel step in the evolution of pathway concepts––the incorporation of the AOP construct into human health research and drug discovery. Our proposed disease AOPs, like AOPs in toxicology, would describe a chain of causally linked key events causing downstream effects at several biological levels and provide clear mechanistic rationales for diagnostic, preventative, and therapeutic interventions in the era of personalized medicine.

The important commonalities between safety science and health research, drug discovery, and clinical translation argue for the relevance of the AOP concept in all these fields. These common features include *a*) human biological pathways whose response continuum encompasses efficacy, adaptation, and adversity; *b*) shared research tools and technologies (e.g., *in vitro* models, analytical approaches, computational modeling); and *c*) the benefits of better-structured and transparent weight-of-evidence decision-making frameworks, whether for chemical safety or drug efficacy, that can integrate all the data inputs.

Our proposed AOPs for human diseases are a natural extension of the AOPs developed in toxicology. The central steps will likely be similar, although the molecular initiating events will be more varied. For example, as well as chemical perturbations, infectious and genetic factors may initiate the disease process. Nevertheless, the principles and basic biology will be shared between disease AOPs and toxicity AOPs, and the related information could be integrated into the existing OECD AOP-KB, including information compiled by several programs designed to leverage big data such as the NIH Big Data to Knowledge initiative (https://datascience.nih.gov/bd2k/), as well as information from existing pathways and bioinformatics databases [e.g., the Kyoto Encyclopedia of Genes and Genomes (http://www.genome.jp/kegg/)] and the gene–disease database DisGeNET (http://www.disgenet.org/).

In the context of disease research and drug discovery, our disease AOP concept would provide a unified framework for describing relevant pathophysiological pathways and networks across multiple biological levels and for encompassing extrinsic and intrinsic causes. Describing these pathways and networks, along with anchoring molecular initiating events with adverse outcomes, our AOP framework would represent a significant advance over existing concepts, such as disease mechanisms that are often studied in isolation and biological pathways or networks (e.g., for cancers) that are invariably considered only at the molecular or cellular levels.

The disease AOP approach would better exploit advanced experimental and computational platforms for knowledge discovery, since the emergence of AOP networks will identify knowledge gaps and steer investigations accordingly. A commitment to build, curate, and disseminate a pathways framework within the biomedical research field would thus provide considerable impetus to base decisions on mechanistic understanding rather than empirical observation, as has been the case in toxicology.

*Advanced human-specific disease models.* In addition to a strategic and integrated knowledge-based exploitation of omics tools and the introduction of the AOP concept, we further propose a strong focus on human-specific models. Advanced human-specific cell- and tissue-based models (e.g., Singh et al. 2011) and next-generation tools are making possible a fuller, dynamic comprehension of disease pathophysiology and a more reliable and cost-effective drug discovery process (Muotri 2015).

Human-induced pluripotent stem cell technology offers unique access to healthy as well as patient- and disease-specific *in vitro* cell models (Bellin et al. 2012). This could help achieve the holy grail of relating disease genotype to phenotype, for example by correlating individual genetic variants with gene expression patterns, disease pathways, and associated outcomes. Models derived from human stem cells have been developed to enhance research into autism spectrum disorders (Marchetto et al. 2010), cardiovascular disease (Zanella et al. 2014), Alzheimer’s disease (Choi et al. 2014), and many other illnesses. In some instances, insights about molecular disease mechanisms and drug effects have emerged from human stem cell systems that were previously missed in nonhuman models (Marchetto et al. 2010; Mitne-Neto et al. 2011).

Human organ-on-a-chip culture devices, combining microfluidics with two- and three-dimensional cell culture, aim to reproduce key architectural, physical, functional, and biochemical features of human organs *in vitro*. Within miniature cell chambers, highly controlled cell culture allows *in vivo*–like interactions between multiple cell types (van de Stolpe and Kauffmann 2015). Identifying and independently varying critical cellular and molecular disease contributors is difficult in animal models, but in microfluidic systems, molecular factors and different cell types can be varied independently and simultaneous measurements of real-time system-level responses become practical (Benam et al. 2015; van de Stolpe and den Toonder 2013). There are already prototype microfluidic models for diseases of the heart, lung, intestine, liver, and kidney and of the vascular, endocrine, musculoskeletal, nervous system, and more (Benam et al. 2015).

Key information is also provided by studies of *ex vivo* biopsied or postmortem human tissue (Zerhouni 2014; Beach 2013) using powerful analytical tools such as next-generation sequencing (Twine et al. 2011), and novel multiplexed fluorescent *in situ* cell and tissue visualization technologies for proteins, DNA, and RNA molecules (Weibrecht et al. 2013) using digital pathology platforms that enable quantification of complex staining patterns. In addition, advanced mass spectrometry techniques can provide high-throughput, comprehensive, and quantitative information about proteins in clinical cell (e.g., tumor biopsies) and biofluid samples (e.g., urine, saliva, or plasma) at high sensitivity (Jimenez and Verheul 2014). Interpreting omics data from healthy and diseased tissues using bioinformatics tools has revealed associations with multiple pathways important in (patho)physiology (Andreev et al. 2012), including information on the status and dynamics of regulatory gene networks and pathways (Tonevitsky et al. 2013). Access to biobanks with well-characterized human tissues, cells, and biofluids from phenotyped patients and controls will also be important. Finally, advanced clinical studies to obtain *in vivo* human information (the true gold standard model) also have much to offer. They may provide new insights into pathology (Rosén et al. 2013; Ledford 2008) and anchor research models of all kinds to real-world illnesses in humans (Koren et al. 2007).

Multidisciplinary data should be integrated and interpreted by means of systems biology tools (van der Sijde et al. 2014). New bioinformatics approaches become even more powerful with the incorporation of cell biology data, and systems biology offers ways to integrate computational and experimental methods at multiple scales from biochemistry through to individual levels (Figure 2). Development and adaptation of integrated software platforms are central to efficient and effective use of data and for predictive computational modeling (Ghosh et al. 2011).

*Toward a new research paradigm.* The key driver for a new paradigm in health research is the slow progress scientists have made in understanding human disease. This has resulted in a lack of success in drug discovery and translation of laboratory findings into effective therapies and in the spiraling investment of resources wasted by late-stage drug failures (Kaitin and DiMasi 2011). There are many reasons for failures in translation to the clinic, but the reliance on animal models, which are limited by species and strain differences and yet continue to dominate decisions throughout the drug discovery and development process, is a key issue which urgently needs to be addressed (Collins 2011; Langley 2014; Pound and Bracken 2014; Seok et al. 2013). The second driver is the emergence of novel scientific tools and models that enable, for the first time, advanced approaches that could revolutionize our understanding and treatment of human disease (Collins 2011).

The transformational potential of 21st-century scientific advances will not be realized if they are simply added to a growing list of existing methods within an outdated 20th-century paradigm of health research and drug discovery. Medical research is now poised to capitalize on the same paradigm shift that is transforming toxicological science, in terms of the overarching framework of research and how data are interpreted and integrated. Toxicology increasingly emphasizes improving prediction by human biology-based models and by focusing on AOPs to exploit systems biology thinking and advanced mathematical modeling. Recognition of the need to change direction to a human-based, multiscale–pathway-focused paradigm is critical, as is confidence in the new approaches. Recognition and confidence are increasingly reflected in major programs such as the U.S. funding commitment in the 2016 budget for a precision medicine initiative involving the omics and a million research volunteers (Collins and Varmus 2015) and, in toxicology, the OECD’s AOP Development Programme (OECD 2013).

## Conclusions

Our proposed new research paradigm, adapted from 21st-century toxicology, would involve the following aspects:

Developing a big picture of human diseases, integrating extrinsic and intrinsic causes, and linking environmental sciences with medical research using systems biology.
Introducing a disease AOP concept, analogous to toxicity AOPs, with the intention of providing a unified framework for describing relevant pathophysiology pathways and networks across multiple biological levels.
Creating a strong focus on advanced human-specific research (*in vitro*, *ex vivo*, *in vivo*, and *in silico*) in place of empirical, animal-based studies.

To accomplish the goals outlined in this article, many questions will need to be considered:

To what extent can existing and emerging human models and tools be applied to replace animal studies?
Where are the knowledge and technology gaps?
How can big data be synthesized into actionable knowledge?
Can computational models effectively bridge the *in vitro–in vivo* divide?
How easy will it be to optimize the derivation of enriched populations of disease-relevant cells from human-induced pluripotent stem cells?

In summary, a new coherent roadmap for medical research promises progress in several areas:

Revealing common disease pathways.
Discovering new and multiple human drug targets.
Improving translation.
Reducing late-stage drug attrition.
Facilitating drug repurposing.
Contributing to the development of personalized medicine.
Achieving more reliable and valid data in faster time frames and at lower costs.

It will take a formidable effort and redeployment of funds (e.g., away from efforts to improve animal models) to achieve the new paradigm of a multiscale–pathways-based, human-centered concept for disease research. We hope this article will help launch a serious discourse among researchers, policymakers, regulatory agencies, and research-funding organizations around the world and encourage those who have already begun to think along these lines. Unless rethinking of the 20th-century research paradigm starts now, benefits to patients from 21st-century scientific and technological advances will be unduly delayed.
